# Supplementary material for: Determination of the Cutoff Frequency of Smoothing Filters for Center of Pressure (COP) Data via Kinetic Energy in Standing Dogs
Source: Sensors (Basel). 2025 Sep 18;25(18):5843. doi: 10.3390/s25185843 (PMC12473882; doi:10.3390/s25185843)
Supplement: Supplementary file 1 [file sensors-25-05843-s001.zip › sensors-3835312-supplementary.pdf]

Table S1: Pairwise Comparison of the different filtered data

					95% Confidence interval for difference[b]	
(I) Filter	(J) Filter	Meandiff (I-J)	Std.-error	Sig.[b]	lower limit	upper limit
UF	6 Hz	-725,833*	37,638	<,001	-831,975	-619,692
	10 Hz	-629,500*	33,502	<,001	-723,976	-535,024
6 Hz	UF	725,833*	37,638	<,001	619,692	831,975
	10 Hz	96,333*	14,164	<,001	56,391	136,276
10 Hz	UF	629,500*	33,502	<,001	535,024	723,976
	6 Hz	-96,333*	14,164	<,001	-136,276	-56,391

Based on estimated marginal resources

*. The difference in mean values is statistically significant at the 0.05 level.

b. Adjustment for multiple comparisons: Bonferroni.
